# Phenolic Compounds in Apple (*Malus* x *domestica* Borkh.): Compounds Characterization and Stability during Postharvest and after Processing

**DOI:** 10.3390/antiox2030181

**Published:** 2013-09-18

**Authors:** Alessandra Francini, Luca Sebastiani

**Affiliations:** BioLabs, Institute of Life Sciences, Scuola Superiore Sant’Anna, Piazza Martiri della Libertà 33, Pisa I-56127, Italy; E-Mail: francini@sssup.it

**Keywords:** anthocyanidins, anthocyanins, flavanols, flavonols, phenolic acids, hydroxycinnamates, phytochemical compounds

## Abstract

This paper summarizes the information on the occurrence of phenolic compounds in apple (*Malus* x *domestica* Borkh.) fruit and juice, with special reference to their health related properties. As phytochemical molecules belonging to polyphenols are numerous, we will focus on the main apples phenolic compounds with special reference to changes induced by apple cultivar, breeding approaches, fruit postharvest and transformation into juice.

## 1. Introduction

In the last few decades the attention of markets to new products, having nutraceutical properties, *i.e.*, able to decrease the risk of diseases, has boosted scientific research to identify and characterize antioxidant properties, molecules in food products, and their derivates. The development of functional foods with health-beneficial properties is one of the main goals of food science research.

One class of molecules that has significant health properties is that of polyphenols. In general terminology, they are considered as a: “*Structural class of mainly natural*, *organic chemicals compounds characterized by the presence of large multiples of phenol structural units*” [1]. In a more strict definition [1], the name “polyphenol” should be used to identify: “*Plant secondary metabolites derived exclusively from the shikimate-derived phenylpropanoid and/or the polyketide pathway(s)*, *featuring more than one phenolic ring and being devoid of any nitrogen-based functional group in their most basic structural expression*”.

Polyphenols are usually divided into several different groups (simple phenols, benzoic acids, phenyl propanoids, and flavonoids) on the basis of the number of carbon atoms in conjunction with the structure of the basic phenolic skeleton [2]. They originate from the plant aromatic pathway, starting with amino acids of the shikimate pathway and culminating in molecules produced by the phenyl propanoid and flavonoid pathways. The coordinated induction-regulation of these pathways leads to the production of several thousand different molecules.

**Figure 1:**
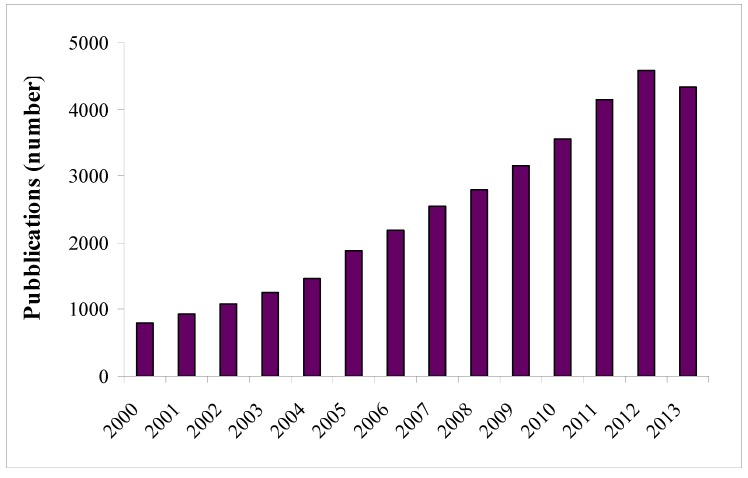
Number of publications, which include polyphenol research, since 2000. Publications registered in the ScienceDirect database [4] where the keyword “polyphenols” is used. The 2013 data are related to the period of January–mid-July.

The general role of phenolic compounds in plant physiology and allelopathy has been acknowledged a long time ago and is still actively studied by plant scientists. More recently, these secondary metabolites, which naturally and abundantly occur in fruits, have been rediscovered by human nutritionists as beneficial molecules and potentially interesting components for the production of functional food (foods that have a potentially positive effect on health beyond basic nutrition). Their positive effects on human health were first proposed in 1936 [3], and the scientific consensus is now common as proven by the remarkable increase in the number of scientific publications where the “polyphenols” term appears (Figure 1).

Several excellent reviews are focused on the health related effects and bioavailability of polyphenols in human nutrition [5,6,7,8,9]. Originally the main health claims for polyphenols were based on their properties as scavengers of free radicals and reactive oxygen species (ROS). However, while polyphenols may have a significant antioxidant *in vitro* activity, the absorption into the human body is more complex [8], and intact forms of complex dietary polyphenols have limited bioavailability. Consequently, the circulating level in plasma is low and a major part of polyphenols persists in the colon where they undergo further metabolic changes before entering the systemic circulation. For such reasons, it is still in doubt if polyphenols can make a significant contribution to free-radical scavenging activity in human as shown for other antioxidant molecules, such as ascorbic acid [10]. However, the cancer preventive effects of green tea polyphenols can arise by induced antioxidative or pro-oxidative effects, and the importance of such effects may depend on the stage of carcinogenesis [11]. These authors suggested that the increased endogenous antioxidant capacity may be more important prior to carcinogen exposure, whereas pro-oxidant cell killing effects may be more important in clearing transformed cells from the body and thus limiting tumour growth. More recently, several dietary polyphenols (caffeic acid, catechin, chlorogenic acid, *etc.*) have been shown to influence epigenetic processes (*i.e.*, heritable changes not encoded in the DNA sequence itself, that play important roles in gene expression regulation) such as DNA methylation processes [7], hystone modification, and microRNA expression [12]. Polyphenol induced epigenetic changes may explain the chemo-preventive roles of these molecules in preventing cancer and other diseases in humans but further studies are needed.

In addition to their health promoting properties in humans, polyphenols are induced in plants under oxidative stress conditions and support the activity of other important cellular antioxidant compounds such as glutathione, α-tocopherol, ascorbic acid, and enzymes such as peroxidase, and superoxide dismutase. Phenolic compounds accumulated in plant organs (roots, stems, leaves, flowers, fruits, *etc*.), according to species characteristics, and are usually more abundant in the epidermal tissue of the organs, such as in the peel of fruit. This preferential localization is set in relation with effect of light on the phenolic metabolism, as well as, with the protective role of phenolic compounds against ultraviolet radiations and other abiotic and biotic stressors [13,14].

The aim of this article is to revise the scientific literature on the biochemical and antioxidant characterization of apple (*Malus* x *domestica* Borkh.) fruit at harvest, and the possible strategies and solutions for maintaining these properties. As phytochemical compounds could undergo relevant modifications during fruit storage and processing in juice, these aspects will be analyzed and discussed.

## 2. Apple

**Figure 2:**
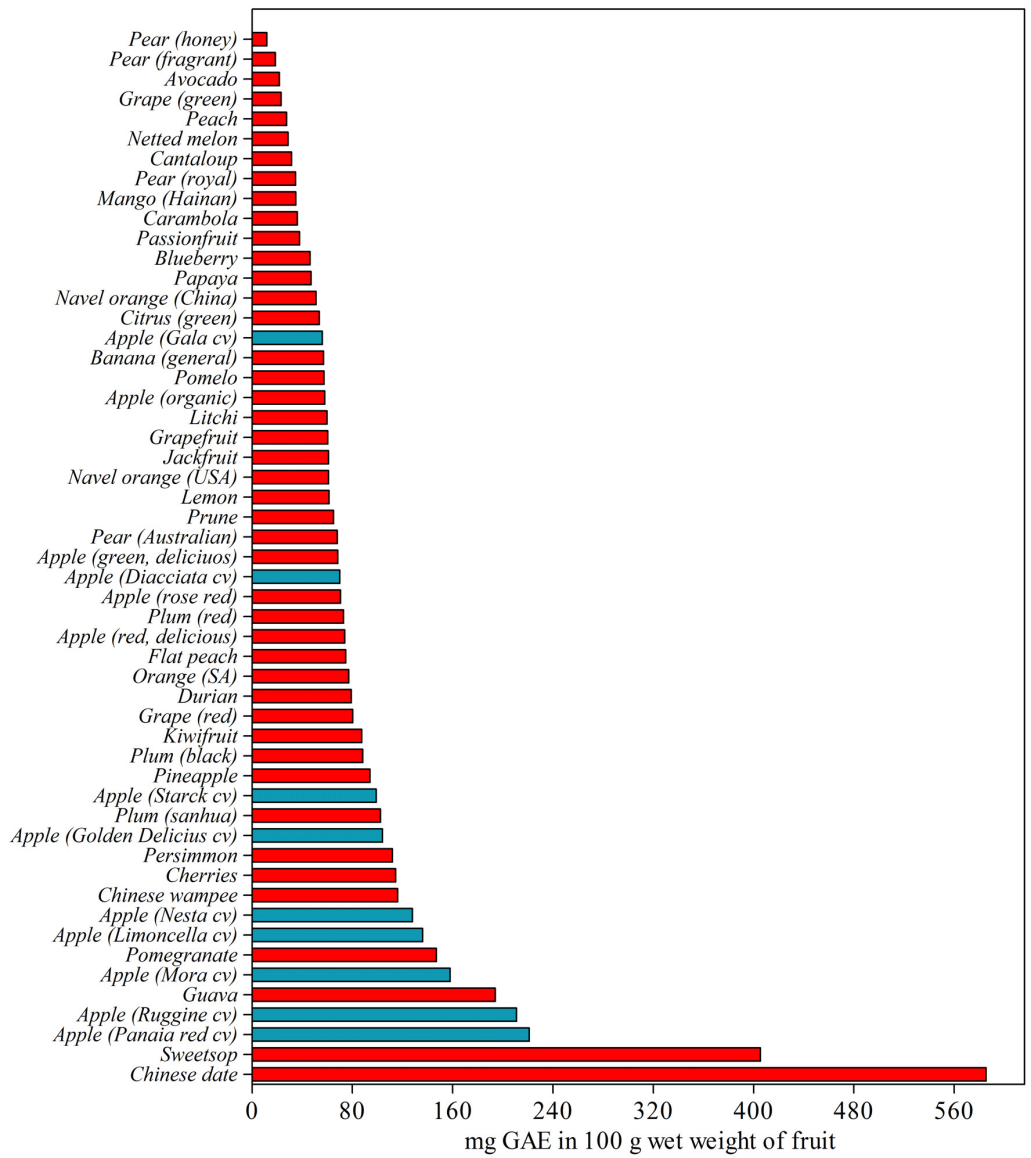
Total phenolic content in different fruits and apple cultivars. Data were re-elaborated from Fu *et al*. [16]—red bars; Iacopini *et al*. [17], and Minnocci *et al*. [18]—blue bars.

Apples are one of the most commonly consumed fruits in the world. In 2011, world apple production was estimated at around 75 millions of tons according to Food and Agriculture Organization stats [15]. Apple are eaten both raw and as processed products, such as cider, juice, and puree. The famous sentence: “*An apple a day keeps the doctor away*!” is what is highly recommended and heavily advertised nowadays to the general public to stay fit and healthy. This claim is due to the high nutraceutical values of the apple’s compounds and to the large abundance and accessibility of this fruit in the market, and, due to postharvest storage technology, fruit shelf life can be extended for up to one year, depending on the variety. Although apples are one of the most consumed fruits in the word it is important to note that they are not those with the greatest phenolic content and antioxidant capacity. In fact, total phenolic contents of 62 fruits using the Folin-Ciocalteu method showed values ranging from 11.88 (*Pyrus communis* L.) to 585.52 (*Ziziphus jujuba* Mill.) mg GAE/100 g of wet weight. In this wide range, apples belonging to green-delicious, red-delicious, and rose-red cultivars showed intermediate values of 68.29, 73.96, and 70.57 mg GAE/100 g of wet weight, respectively [16]. Specific studies aimed at comparing total polyphenols in commercial and ancient apple cultivars were performed by Iacopini *et al*. [17] and Minnocci *et al*. [18]. These studies showed that cultivar effects can be relevant as total polyphenol content range between 56 and 221 mg GAE/100 g of wet weight in Gala and Panaia red cultivars, respectively (Figure 2). These results prove that the genetic variability within apple germplasm can provide significant genetic variation for polyphenol traits.

### 2.1. Phenolic Compounds in Apple

Apples contain a variety of phenolic compounds [19] and, using liquid chromatography-mass spectrometry (LC-MS) or gas chromatography mass spectrography (GC-MS) analysis methods, it is possible to detect several polyphenolic molecules, such as (+)-catechin and (−)-epicatechin (flavan-3-ols or flavanols), phloridzin (dihydrochalcone glycosides), quercetin (flavonols), cyaniding (anthocyanidins), cyanidin-3-*O*-galactoside (anthocyanins), chlorogenicacid (phenolic acids), and hydroxycinnamates (p-coumaric acid) [20,21]. In general, the polyphenolic contents per fruit ranges between 19.6 and 55.8 (flavan-3-ols), 17.7–33.1 (flavonols), and 10.6–80.3 (chlorogenic acid) mg per apple; the lowest values were recorded for phloridzin (1.0–9.3 mg per apple) and anthocyanin (0.1–6.5 mg per apple) [22].

Total phenolic, flavonoid, and anthocyanin contents in four apple varieties (Rome Beauty, Idared, Cortland, and Golden Delicious) were compared in flesh and peel [23]. The total phenolic contents of the peel were highest in Idared and Rome Beauty (588.9 and 500.2 mg of GAE/100 g of peel, respectively) and drop to 75.7 and 93.0 (mg of GAE/100 g of flesh, respectively). For flavonoids, the Idared peel contains 303.2 mg of catechin equivalents/100 g, corresponding to six times higher concentrations than in flesh. Anthocyanins were detected only in peel, ranging from trace amount in Golden Delicious (yellow/green peel) to 26.8 mg of cyanidin 3-glucoside equivalents/100 g of peel in Idared. The cumulative sum of polyphenols (flavanols, flavonols, phloridzin, procyanidin, chlorogenic acid, anthocyanin), expressed on a per apple basis, was determined on 10 apple cultivars coming from different regions and growing conditions in New Zealand, and varies between 263.1 and 97.7 mg of total phenolics per apple in Pacific Queen to Cox’s Orange cultivar, respectively [22].

**Figure 3:**
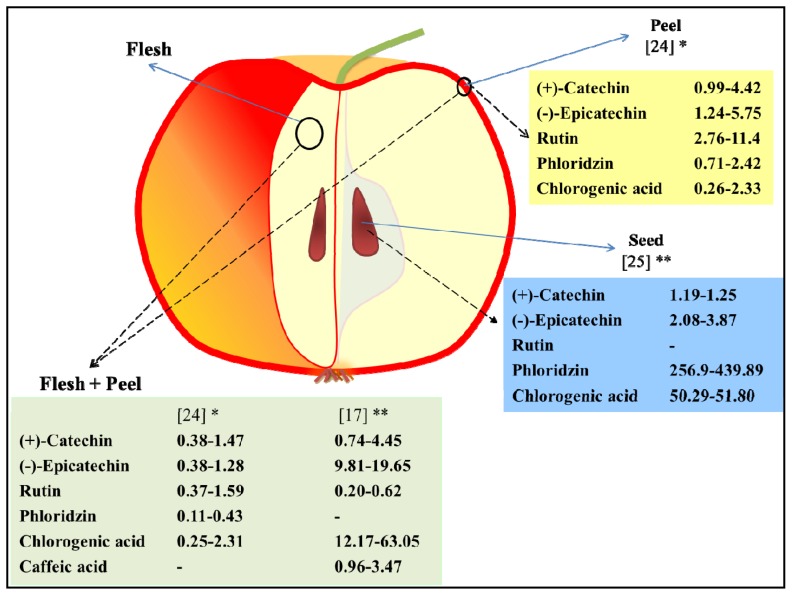
Polyphenol molecule concentrations ranges in seed, peel and peel + flesh. Data were re-elaborated form, Łata *et al*. [24], Duda-Chodak *et al*. [25], and Iacopini *et al*. [17]. * mg/g DW; ** mg/100 g FW; - not measured.

Deeper characterization of apples polyphenol molecules associated with their localization in peel, flesh, and seeds, prove that peel, and also seeds, are rich in these compounds [22,24,25]. A schematic representation of concentration ranges of specific polyphenols in peel [24], peel and flesh [15,22] and seeds [25], is presented in Figure 3. In addition, seeds are usually discharged when eating apple fruit, the peel (which represents a small portion of the whole fruit weight) can provide a significant fraction of the phenolics, becoming an important donor of these compounds as confirmed by the literature [22,24]. Flesh and peel values for (+)-catechin, (−)-epicatechin, phloridzin, quercetin, cyanidin-3-*O*-galactoside ranges between 0.45–3.4, 5.18–18.40, 0.64–9.11, 0.10–0.22, and not detectable-3.11 mg per 100 g fresh weight, respectively [21].

### 2.2. Health Benefits of Apple Phenolics

Evidence suggests that a diet rich in apples may reduce the risk of diseases. For polyphenols, apples are fruits for which numerous data are available [5], and each polyphenol molecule might have specific health benefits. For example, the non-glycosilated form of phlorizin, phloretin, has been shown to influence epigenetic processes, heritable changes not encoded in the DNA sequence itself that play an important role in gene expression regulation in breast cancer cells [26]. Other polyphenols, such as quercetin, are efficient inhibitors of sulfotransferases [27,28], and may change the activity of thyroid hormones, steroids, and catecholamines [29].

Multiple and important mechanisms of cancer are affected by apple components, more specifically by oligomeric procyanidins. These effects embrace *in vitro* antimutagenic activity, modulation of carcinogen metabolism, antioxidant activity, anti-inflammatory mechanisms, modulation of signal transduction pathways, anti-proliferative, and apoptosis-inducing activity, as well as novel mechanisms on epigenetic events and innate immunity [30].

Apple polyphenols may also have beneficial effects on outcomes related to Alzheimer’s disease. Apple juice may work in cognitive decline of normal aging suppressing over expression of presenilin-1, which is linked to the production of amyloid *b* peptide, a marker of Alzheimer’s [31].

The phloretin-*O*-glycosides, phloretin-2′-*O*-glucoside and phloretin-2′-*O*-(2″-*O*-xylosyl) glucoside, are thought to be unique to apples and apple products. Their role on inhibition of sodium-dependent glucose transporters in the intestinal lumen, is proven with special emphasis for phloretin-2′-*O*-glucoside [32]. By decreasing the absorption of glucose, phloretin-2′-*O*-glucoside may, therefore, reduce post-prandial blood glucose levels, and it is thought this action may be beneficial to the treatment of diabetes mellitus. The authors suggested an interesting role for apple polyphenols related to glucose control in diabetes [33].

The potential role of apple polyphenols to reduce or prevent injury to gastric mucosa by drugs was investigated in a combined study using cell and animal models. The apple extract tested that was highest in chlorogenic acid protected cells from oxidative damage, moreover, the fraction highest in catechin also protected cells from oxidative damage in a dose-dependent manner [34].

In animal models, apples have been shown to prevent skin, mammary, and colon carcinogenesis, while in epidemiological observations indicated that regular consumption of one or more apples a day may reduce the risk for lung and colon cancer [35,36]. An Italian research group studying the effects of ten weeks of fresh Annurca apple intake in aged rats, found that regular apple consumption in aged rats restored synaptic function to the level of younger animals [37].

Apples fruit, as well apple juice and other derivate products, have a rich phytochemical profile suggesting their potential to affect the health of the populations consuming them [36]. A hospital-control study including over 6000 participants from various geographical areas in Italy examined the association between fresh apple intake and risk of cancer [38]. Data were based on interviews of dietary intake in the two years prior to diagnosis. It was found that consuming one, or more than one, apple per day was associated with a reduction in risk of cancer compared to consumption of less than one apple per day. Furthermore, a hospital-based case-control study carried out in Poland showed a significant beneficial effect of apple consumption (daily number of apple servings) on the risk of colorectal cancer. The dietary interviews focused on food-frequency and quantity, and proved that apples were the most frequent fruit consumed in the study population. Out of several types of fruits in the study, which included citrus, berries, and stone fruits, apple was the only specific type of fruit associated with a significant reduction (63%) of colorectal cancer risk [39].

Finally, an evaluation of cardiovascular protective effect of different apple varieties on rats proved that catechin, epicatechin, and procyanidin B1 polyphenols are the major compounds responsible for the cholesterol lowering ability of apples [40].

### 2.3. Stability of Phenolics in Apple Fruit during Postharvest and in Juice

In apple, as in other fruits, the variability of polyphenols profile is influenced by cultivar, ripening stage, growing season, environmental factors, geographic region, production techniques, and storage conditions [22,41,42]. Due to the evidence that polyphenol content is largely cultivar dependent, different results have been described in the literature dealing with apple fruit postharvest storage. Carbone and collaborators [43] studied the influence of genotype, tissue type, and cold storage on bioactive compounds of different apple cultivars (Golden clone B, Fuji clone Kiku8, and Braeburn clone Hillwell). Authors showed that total phenol content was dramatically reduced after cold storage (1 °C for three months) in flesh (50%) and peels (20%) of apple Hillwell, but not in the other cultivars. More complex results were found by Begić-Akagićand collaborators [44]. These authors showed that apple phenolic content in relation to storage time (60 days at 1 °C) slightly decreased in three common apple cultivars (Topaz, Pinova, and Pink Lady) and three autochthonous apple cultivars (Ruzmarinka, Ljepocvjetka, and Paradija) from Bosnia and Herzegovina.

Shelf life at ambient temperature (20 °C for two weeks) proved that phenol contents of apple Golden Delicious, Pinova, Mairac, and Honey crisp cultivars did not change and an increase of these compounds was even observed for Wellant cultivar. A significant decrease (50%) in phenol content was measured in Topazin comparison to that measured at harvest [45]. Finally, Napolitano and collaborators [46] observed an increase, in the flesh of Annurca (a local Italian apple), of catechin and phloridzin related to antioxidant activity during cold storage.

Such remarkable variability determines a difficult comparison among data, so we avoid a deep review of this literature and focus our attention on the main quantitative and qualitative changes of phenolic compounds during fresh fruit transformation and storage processes. The different concentrations of phenols in apple fruit tissues affects processed products phenolic quality and it has been well demonstrated that, during processing of primary products, the antioxidant capacity of apple may be lost. In particular, the general loss of antioxidant capacity starts as soon as the integrity of the cell is broken and enzymes, such as esterases, glycosidases, and decarboxylases, may start to catalyse the transformations and degradations of phenolic compounds [47].

For processed apple products, the most popular is apple juice. During its production, only a fraction of phenolic compounds are extracted, while the other remains in the pomace [48]. Due to the fact that peel and seeds are discharged during juice production, phenolic compounds such as quercetin glycosides and dihydrochalcones, are found in small amounts in apple juice [49]. Apple pomace is the “waste” product generated during apple juice processing. Lu and Foo [50] investigated apple pomace as a potential source of natural polyphenols for use as dietary or food antioxidants. In this study, apple Gala polyphenols purified from pomace exhibited high antioxidant activities: three-times the DPPH-scavenging and 10–30 times superoxide-scavenging activities of vitamins C or E. According to the taste of consumers, apples are processed in clear apple juices but during this process many components with high antioxidant potential are lost [49]. In particular, (−)-epicatechin and procyanidins are removed during the clarification process of apple juices. On the contrary in cloudy juices the dark products of oxidation are not removed from the juice [50].

After processing, the apple juice is usually stored. Storage at room temperature (25 °C) of apple juice, for nine months, resulted in a 60% loss of quercetin and a total loss of procyanidins, despite the fact that polyphenols are more stable in fruit juices than vitamin C [51,52].

### 2.4. Improving Phenolics Profile in Apple Fruit: From Germplasm Recovery and Classical Breeding to GM Plants

The massive spread of commercial apples in the last decades has resulted in the disappearance from the market of the local/ancient cultivars with the risk of losing some health related quality attributes of these fruits. In order to analyze and recover this germplasm, many researchers are aiming to evaluate the antioxidant properties and phenolic profile of these ancient varieties.

Iacopini and collaborators [17] studied the phenolic content and the antioxidant properties of ancient and commercial varieties grown in Italy. Four ancient apple varieties (Nesta, Mora, Panaia, and Ruggine) and two commercial varieties (Golden delicious and Stark delicious) were analyzed and their antioxidant activities were compared using peroxynitrite (ONOO^−^) and dipheny l-2,4,6-trinitrophenyl-iminoazanium (DPPH) assays. Analyses were performed pooling peel and flesh together. Golden delicious was found to be the variety with the lowest content of almost all the phenolic compounds, whereas the old varieties Ruggine and Panaia contained the highest levels. The antioxidant activity was positively correlated with the total polyphenolics concentration and with the concentration of the principal phenolic compounds present in apple extract; catechin, epicatechin, and chlorogenic acid. These results suggest the relevance of ancient apple germplasm for providing fruit with high polyphenolic content and antioxidant scavenging properties (Figure 2) [17]. Similar data were obtained in two late-bearing apple cultivars, Diacciata and Limoncella, when compared with two modern commercial cultivars (Gala and Golden Delicious) [18]. In this study the phenolic concentration in Limoncella was particularly high (around twice that of Diacciata and almost three times higher than Gala) confirming the importance of maintaining and screening these ancient germplasm as potential source of genes for apple breeding program by which polyphenols may be altered in apple fruit.

Total and individual polyphenols in apple cultivars have been shown to vary, but few data are available on the variability outside the specie commonly cultivated. The juice of over 300 non-commercial genotypes derived from 20 species of *Malus*, highlighting a 400-fold variation in total fruit polyphenol concentrations [53]. The highest polyphenol concentrations were found in large-sized *Malus sieversii* fruit, and authors recommended this species as genetic parental material for future breeding program.

The availability of the apple genome sequence [54] has caused a boost of apple genetics and genomic research by providing new tools for identifying genes and other functional elements [55]. In several plant species, pigmentation is controlled by the relative amounts of anthocyanins, chlorophyll, and carotenoids pigments. As these have potential positive effects on human health, fruit breeding has to exploit germplasm collections to develop new varieties with improved pigmentation, such as the breeding of red-fleshed apples with elevated concentrations of anthocyanins [56]. However, this approach involves crossing red-fleshed wild apples relatives (form the center of origin of apple in Central Asia) with modern white fleshed commercial varieties and requires many backcrosses to eliminate unwanted characteristics, such as poor taste, texture, or storage traits, from the cross derived progeny. Thanks to molecular biology and genomic data, alternative approaches are now exploring the direct integration of the dominant red-flesh MYB allele [57] into modern high-quality commercial apple cultivar via a transgenic/cisgenic approach [58]. Using this biotechnological solution, Espley and collaborators [58] raised the polyphenolic content of Royal Gala apple by genetic engineering of the anthocyanin pathway using the apple transcription factor MYB10. The transgenic apples had very high concentrations of foliar, flower, and fruit anthocyanins: from 58.2 to 561.2–855.8 mg·kg^−1^ dry weight in the fruit peel, and from not detected to 565.2–208.0 mg kg^−1^ dry weight in the fruit cortex (Royal Gala and MYB10 transgenic lines, respectively). Notably, no negative taste attributes were associated with the elevated anthocyanins indicating that red-fleshed apples retain consumer expectations of flavor, adding a potential health enhancement. In future, it may be possible to genetically modify elite apple genotypes for this and other specific polyphenolic traits while retaining their highly valuable agronomic and quality properties.

## 3. Conclusions

On the basis of the data related to apple polyphenols that we reviewed in this paper, some schematic conclusion could be given:

- literature on apple polyphenols properties suggest their potential use in preventing several chronic diseases in humans.
- Deeper knowledge on the specific molecular mechanisms of action (*i.e.*, epigenetic modifications) of apple polyphenols in human disease is necessary.
- Phenolic compound characterization in whole apple fruit is well established, while their fate during transformation in juice need to be improved in order to better clarify the losses of these compounds and suitable strategies for their optimal conservation.
- The high variation on the localization of polyphenols composition in the fruit peel and flesh among cultivars and *Malus* species can be very useful for breeding program and underline the importance of germplasm conservation strategies.
- Genomic revolution and biotechnological applications will boost genetic improvement of elite apple genotypes by enabling the introduction of highly specific polyphenolic traits.

## References

[1] Quideau S., Deffieux D., Douat-Casassus C., Pouysegu L. (2011). Plant Polyphenols: Chemical properties, biological activities, and synthesis. Angew. Chem. Int..

[2] Harborne J.B. (1964). Biochemistry of Phenolic Compounds.

[3] Bensath A., Ruysnyak T., Szent-Györgii A. (1936). Vitamine nature of flavones. Nature.

[4] ScienceDirect Database. www.sciencedirect.com.

[5] Manach C., Scalbert A., Morand C., Rémésy C., Jiménez L. (2004). Polyphenols: Food sources and bioavailability. Am. J. Clin. Nutr..

[6] Pandey K.B., Rizvi S.I. (2009). Plant polyphenols as dietary antioxidants in human health and disease. Oxid. Med. Cell Longev..

[7] Link A., Balaguer F., Goel A. (2010). Cancer chemoprevention by dietary polyphenols: Promising role for epigenetics. Biochem. Pharmacol..

[8] Van Duynhovena J., Vaughana E.E., Jacobsa D.M., Kempermana R.A., van Velzena E.J.J., Grossa G., Rogera L.C., Possemiersd S., Smildec A.K., Doréb J. (2011). Metabolic fate of polyphenols in the human superorganism. Proc. Natl. Acad. Sci. USA.

[9] Visioli F., Alarcón De La Lastra C., Andres-Lacueva C., Aviram M., Calhau C., Cassano A., D’Archivio M., Faria A., Favé G., Fogliano V. (2011). Polyphenols and human health: A prospectus. Crit. Rev. Food Sci. Nut..

[10] Frei B., England L., Ames B.N. (1989). Ascorbate is an outstanding antioxidant in human blood plasma. Proc. Nat. Acad. Sci. USA.

[11] Lambert J.D., Elias R.J. (2010). Antioxidant and pro-oxidant activities of green tea polyphenols: A role in cancer prevention. Arch. Biochem. Biophys..

[12] Pan M.H., Lai C.S., Wu J.C., Ho C.T. (2013). Epigenetic and diseases targets by polyphenols. Curr. Pharm. Des..

[13] Winkel-Shirley B. (2002). Biosynthesis of flavonoids and effects of stress. Curr. Opin. Plant Biol..

[14] Lattanzio V., Lattanzio V.M.T., Cardinali A., Imperato F. (2006). Phytochemistry: Role of Phenolics in the Resistance Mechanisms of Plants against Fungal Pathogens and Insects. Phytochemistry: Advances in Research.

[15] Food and Agriculture Organization (FAO). http://faostat3.fao.org/home/index.html.

[16] Fu L., Xu B.T., Xu X.R., Gan R.Y., Zhang Y., Xia E.Q., Li H.B. (2011). Antioxidant capacities and total phenolic contents of 62 fruits. Food Chem..

[17] Iacopini P., Camangi F., Stefani A., Sebastiani L. (2010). Antiradical potential of ancient Italian apple varieties of *Malus* x *domestica* Borkh. In a peroxynitrite-induced oxidative process. J. Food Comp. Anal..

[18] Minnocci A., Iacopini P., Martinelli F., Sebastiani L. (2010). Micromorphological, biochemical, and genetic characterization of two ancient, late-bearing apple varieties. Eur. J. Hort. Sci..

[19] Scalbert A., Williamson G. (2000). Dietary intake and bioavailability of polyphenols. J. Nutr..

[20] Cuthbertson D., Andrews P.K., Reganold J.P., Davies N.M., Lange B.M. (2012). Utility of metabolomics toward assessing the metabolic basis of quality traits in apple fruit with an emphasis on antioxidants. J. Agric. Food Chem..

[21] Vrhovsek U., Rigo A., Tonon D., Mattivi F. (2004). Quantitation of polyphenols in different apple varieties. J. Agric. Food Chem..

[22] McGhie T.K., Hunt M., Barnet L.E. (2005). Cultivar and growing region determine the antioxidant polyphenolic concentration and composition of apples grown in New Zealand. J. Agric. Food Chem..

[23] Wolfe K., Wu X., Liu R.H. (2003). Antioxidant activity of apple peels. J. Agric. Food Chem..

[24] Łata B., Trampczynska A., Paczesna J. (2009). Cultivar variation in apple peel and whole fruit phenolic composition. Sci. Hort..

[25] Duda-Chodak A., Tarko T., Tuszyński T. (2011). Antioxidant activity of apples—An impact of maturity stage and fruit part. Sci. Pol. Technol. Aliment..

[26] Paluszczak J., Krajka-Kuzniak V., Baer-Dubowska W. (2010). The effect of dietary polyphenols on the epigenetic regulation of gene expression in MCF7 breast cancer cells. Toxicol. Lett..

[27] Otake Y., Nolan A.L., Walle U.K., Walle T. (2000). Quercetin and resveratrol potently reduce estrogen sulfotransferase activity in normal human mammary epithelial cells. J. Steroid Biochem. Mol. Biol..

[28] Marchetti F., de Santi C., Vietri M., Pietrabissa A., Spisni R., Mosca F., Pacifici G.M. (2001). Differential inhibition of human liver and duodenum sulphotransferase activities by quercetin, a flavonoid present in vegetables, fruit and wine. Xenobiotica.

[29] Coughtrie M.W., Sharp S., Maxwell K., Innes N.P. (1998). Biology and function of the reversible sulfation pathway catalysed by human sulfotransferases and sulfatases. Chem. Biol. Interact..

[30] Gerhauser C. (2008). Cancer chemopreventive potential of apples, apple juice, and apple components. Planta Med..

[31] Chan A., Shea T. (2009). Dietary supplementation with apple juice decreases endogenous amyloid-beta levels in murine brain. J. Alzheimer’s Dis..

[32] Johnston K., Clifford M., Morgan L. (2002). Possible role for apple juice phenolic compounds in the acute modification of glucose tolerance and gastrointestinal hormone secretion in humans. J. Sci. Food Agric..

[33] Marks S.C., Mullen W., Borges G., Crozier A. (2009). Absorption, metabolism, and excretion of cider dihyrochalcones in healthy humans and subjects with an ileostomy. J. Agric. Food Chem..

[34] Graziani G., D’Argenio G., Tuccillo C., Loguercio C., Ritieni A., Morisco F., del Vecchio B., Fogliano V., Romano M. (2005). Apple phenol extracts prevent damage to human gastric epithelial cells *in vitro* and to rat gastric mucosa *in vivo*. Gut.

[35] Boyer J., Liu R.H. (2004). Apple phytochemicals and their health benefits. Nutr. J..

[36] Hyson D.A. (2011). A comprehensive review of apples and apple components and their relationship to human health. Am. Soc. Nutr. Adv. Nutr..

[37] Viggiano A., Monda M., Turco I., Incarnato L., Vinno V., Viggiano E., Baccari M., de Luca B. (2006). Annurca apple-rich diet restores long-term potentiation and induces behavioural modifications in aged rats. Exp. Neurol..

[38] Gallus S., Talamini R., Giacosa A., Montella M., Ramazzotti V., Franceschi S., Negri E., La Vecchia C. (2005). Does an apple a daykeep the oncologist away?. Ann. Oncol..

[39] Jedrychowski W., Maugeri U., Popiela T., Kulig J., Sochacka-Tatara E., Pac A., Sowa A., Musial A. (2010). Case-control study on beneficial effect of regular consumption of apples on colorectal cancer risk in a population with relatively low intake of fruits and vegetables. Eur. J. Cancer Prev..

[40] Serra A.T., Rocha J., Sepodes B., Matias A.A., Feliciano R.P., de Carvalho A., Bronze M.R., Duarte C.M., Figueira M.E. (2012). Evaluation of cardiovascular protective effect of different apple varieties—Correlation of response with composition. Food Chem..

[41] Tsao R., Yang R., Xie S., Sockovie E., Khanizadeh S. (2005). Which polyphenolic compounds contribute to the total antioxidant activities of apple?. J. Agric. Food Chem..

[42] Duda-Chodak A., Tarko T., Satora P., Sroka P., Tuszyński T. (2010). The profile of polyphenols and antioxidant properties of selected apple cultivars grown in Poland. J. Fruit Ornam. Plant Res..

[43] Carbone K., Giannini B., Picchi V., Scalzo R.L., Cecchini F. (2011). Phenolic composition and free radical scavenging activity of different apple varieties in relation on the cultivar, tissue type and storage. Food Chem..

[44] Begić-Akagić A., Spaho N., Oručević S., Drkenda P., Kurtović M., Gaši F., Kopjar M., Piližota V. (2011). Influence of cultivar, storage time, and processing on the phenol content of cloudy apple juice. Croat. J. Food Sci. Technol..

[45] Matthes A., Schmitz-Eiberger M. (2009). Polyphenol content and antioxidant capacity of apple fruit: Effect of cultivar and storage conditions. J. Appl. Bot. Food Qual..

[46] Napolitano A., Cascone A., Graziani G., Ferracane R., Scalfi L., di Vaio C., Ritieni A., Fogliano V. (2004). Influence of variety and storage on the polyphenol composition of apple flesh. J. Agric. Food Chem..

[47] Cheynier V. (2005). Polyphenols in foods are more complex than often thought. Am. J. Clin. Nutr..

[48] Van der Sluis A.A., Dekker M., van Boekel M.A.J.S. (2005). Activity and concentration of polyphenolic antioxidants in apple juice. Stability during storage. J. Agric. Food Chem..

[49] Markowski J., Płocharski W. (2006). Determination of phenolic compounds in apples and processed apple products. J. Fruit Ornam. Plant Res..

[50] Lu Y., Foo L.Y. (2000). Antioxidant and radical scavenging activities of polyphenols from apple pomace. Food Chem..

[51] Spanos G.A., Wrolstad R.E., Heatherbell D.A. (1990). Influence of processing and storage on the phenolic composition of apple juice. J. Agric. Food Chem..

[52] Miller N.J., Diplock A.T., Rice-Evans C.A. (1995). Evaluation of the total antioxidant activity as a marker of the deterioration of apple juice on storage. J. Agric. Food Chem..

[53] Volz R.K., McGhie T.K. (2011). Genetic variability in apple fruit polyphenol composition in *Malus* x *domestica* and *Malus sieversii* germplasm grown in New Zealand. J. Agric. Food Chem..

[54] Velasco R., Zharkikh A., Affourtit J., Dhingra A., Cestaro A., Kalyanaraman A., Fontana P., Bhatnagar S.K., Troggio M., Pruss D. (2010). The genome of the domesticated apple (*Malus* x *domestica* Borkh.). Nat. Genet..

[55] Troggio M., Gleave A., Salvi S., Chagné D., Cestaro A., Kumar S., Crowhurst R.N., Gardiner S.E. (2012). Apple, from genome to breeding. Tree Genet. Gen..

[56] Volz R., Oraguzie N., Whitworth C., How N., Chagné D., Carlisle C., Gardiner S. (2009). Red flesh breeding in apple-progress and challenges. Acta Hort..

[57] Espley R.V., Brendolise C., Chagne D., Kutty-Amma S., Green S., Volz R., Putterill J., Schouten H.J., Gardiner S.E., Hellens R.P. (2009). Multiple repeats of a promoter segment causes transcription factor autoregulation in red apples. Plant Cell.

[58] Espley R.V., Bovy A., Bava C., Jaeger S.R., Tomes S., Norling C., Crawford J., Rowan D., McGhie T.K., Brendolise C. (2013). Analysis of genetically modified red-fleshed apples reveals effects on growth and consumer attributes. Plant Biotechnol. J..

